# Supplementary material for: Hot spot prediction in protein-protein interactions by an ensemble system
Source: BMC Syst Biol. 2018 Dec 31;12(Suppl 9):132. doi: 10.1186/s12918-018-0665-8 (PMC6311905; doi:10.1186/s12918-018-0665-8)
Supplement: Supplementary file 2 — All relevant MATLAB code. A simple Matlab implement of our predictor. (ZIP 19,742 kb) [file 12918_2018_665_MOESM2_ESM.zip › Additional file 2/Read ASA/ASA Property/SKEMPI/1DVFC.txt]

 NetSurfP - Protein Surface Accessibility and Secondary Structure Predictions

  

|  |  |  |
| --- | --- | --- |
|  |  | NetSurfP - Protein Surface Accessibility and Secondary Structure Predictions  Technical University of Denmark |

  


---

```
# For publication of results, please cite:
# A generic method for assignment of reliability scores applied to solvent accessibility predictions.
# Bent Petersen, Thomas Nordahl Petersen, Pernille Andersen, Morten Nielsen and Claus Lundegaard
# BMC Structural Biology 2009, 9:51 doi:10.1186/1472-6807-9-51
#
# Column 1: Class assignment - B for buried or E for Exposed - Threshold: 25% exposure, but not based on RSA
# Column 2: Amino acid
# Column 3: Sequence name
# Column 4: Amino acid number
# Column 5: Relative Surface Accessibility - RSA
# Column 6: Absolute Surface Accessibility
# Column 7: Z-fit score for RSA prediction
# Column 8: Probability for Alpha-Helix
# Column 9: Probability for Beta-strand
# Column 10: Probability for Coil
E D  1DVFC                  1    0.840 121.030   0.699   0.003   0.003   0.994
E I  1DVFC                  2    0.299  55.241  -0.390   0.004   0.514   0.481
E Q  1DVFC                  3    0.516  92.175   1.141   0.002   0.816   0.182
B L  1DVFC                  4    0.151  27.648   0.575   0.002   0.816   0.182
E T  1DVFC                  5    0.464  64.371   0.338   0.003   0.718   0.279
B Q  1DVFC                  6    0.142  25.361   0.190   0.004   0.420   0.576
E S  1DVFC                  7    0.427  50.044   0.048   0.004   0.085   0.910
E P  1DVFC                  8    0.483  68.552  -0.978   0.058   0.017   0.925
E S  1DVFC                  9    0.521  61.061  -1.243   0.053   0.043   0.903
E S  1DVFC                 10    0.382  44.829  -1.139   0.066   0.296   0.638
B L  1DVFC                 11    0.206  37.682   0.232   0.023   0.655   0.322
E S  1DVFC                 12    0.429  50.279  -0.422   0.021   0.756   0.223
B A  1DVFC                 13    0.253  27.936   0.174   0.022   0.552   0.426
E S  1DVFC                 14    0.434  50.900   0.464   0.022   0.359   0.619
E L  1DVFC                 15    0.514  94.187   0.317   0.004   0.085   0.910
E G  1DVFC                 16    0.626  49.290  -1.201   0.005   0.045   0.951
E D  1DVFC                 17    0.416  59.888   0.621   0.004   0.138   0.858
E R  1DVFC                 18    0.547 125.355   1.264   0.003   0.718   0.279
B V  1DVFC                 19    0.074  11.420  -0.064   0.001   0.959   0.040
E T  1DVFC                 20    0.416  57.741   1.251   0.001   0.959   0.040
B I  1DVFC                 21    0.049   9.009   0.386   0.001   0.959   0.040
E S  1DVFC                 22    0.347  40.727   0.638   0.001   0.900   0.099
B C  1DVFC                 23    0.027   3.819   0.265   0.001   0.900   0.099
E R  1DVFC                 24    0.411  94.165   0.941   0.002   0.816   0.182
B A  1DVFC                 25    0.232  25.522  -1.248   0.003   0.718   0.279
B S  1DVFC                 26    0.347  40.704  -1.257   0.004   0.514   0.481
E Q  1DVFC                 27    0.461  82.281  -1.812   0.004   0.138   0.858
E D  1DVFC                 28    0.436  62.756  -2.510   0.018   0.047   0.935
E I  1DVFC                 29    0.457  84.489  -2.034   0.018   0.047   0.935
E S  1DVFC                 30    0.342  40.129  -1.396   0.018   0.088   0.893
B N  1DVFC                 31    0.264  38.708  -0.616   0.021   0.279   0.699
B Y  1DVFC                 32    0.101  21.541   0.616   0.023   0.655   0.322
B L  1DVFC                 33    0.027   5.017   0.324   0.018   0.846   0.136
B N  1DVFC                 34    0.070  10.307   0.282   0.018   0.846   0.136
B W  1DVFC                 35    0.033   7.840  -0.012   0.018   0.846   0.136
B Y  1DVFC                 36    0.042   9.040   0.431   0.018   0.846   0.136
B Q  1DVFC                 37    0.141  25.111   0.768   0.021   0.756   0.223
B Q  1DVFC                 38    0.084  14.967   0.440   0.021   0.451   0.528
E K  1DVFC                 39    0.370  76.130   1.733   0.004   0.085   0.910
E P  1DVFC                 40    0.339  48.175   0.035   0.005   0.015   0.979
E D  1DVFC                 41    0.767 110.554  -1.570   0.003   0.003   0.994
E G  1DVFC                 42    0.492  38.736   0.074   0.018   0.019   0.964
E T  1DVFC                 43    0.350  48.587  -0.085   0.018   0.047   0.935
B V  1DVFC                 44    0.082  12.573   0.361   0.021   0.279   0.699
B K  1DVFC                 45    0.253  52.063   0.560   0.021   0.756   0.223
B L  1DVFC                 46    0.102  18.603   0.647   0.011   0.918   0.071
B L  1DVFC                 47    0.023   4.248   1.056   0.011   0.918   0.071
B I  1DVFC                 48    0.021   3.885   0.640   0.001   0.959   0.040
B Y  1DVFC                 49    0.100  21.327   0.244   0.001   0.959   0.040
B Y  1DVFC                 50    0.107  22.951  -0.563   0.001   0.900   0.099
B T  1DVFC                 51    0.168  23.357  -0.548   0.004   0.514   0.481
B S  1DVFC                 52    0.234  27.413  -1.250   0.004   0.197   0.799
E R  1DVFC                 53    0.646 147.980  -1.414   0.004   0.085   0.910
E L  1DVFC                 54    0.454  83.182  -0.435   0.005   0.015   0.979
E H  1DVFC                 55    0.589 107.121  -1.943   0.018   0.019   0.964
E S  1DVFC                 56    0.717  84.068  -1.502   0.018   0.047   0.935
E G  1DVFC                 57    0.570  44.867  -1.446   0.018   0.047   0.935
E V  1DVFC                 58    0.316  48.523  -0.186   0.005   0.045   0.951
E P  1DVFC                 59    0.595  84.402  -0.911   0.004   0.085   0.910
E S  1DVFC                 60    0.352  41.266  -1.926   0.004   0.138   0.858
B R  1DVFC                 61    0.255  58.464  -0.460   0.004   0.616   0.381
B F  1DVFC                 62    0.062  12.363  -0.711   0.001   0.900   0.099
B S  1DVFC                 63    0.177  20.803  -0.723   0.001   0.900   0.099
B G  1DVFC                 64    0.148  11.632  -2.520   0.001   0.900   0.099
E S  1DVFC                 65    0.411  48.181  -0.154   0.002   0.816   0.182
E G  1DVFC                 66    0.434  34.148  -0.820   0.004   0.514   0.481
E S  1DVFC                 67    0.561  65.796  -1.675   0.004   0.197   0.799
E G  1DVFC                 68    0.644  50.699  -1.092   0.005   0.045   0.951
E T  1DVFC                 69    0.413  57.325  -0.322   0.005   0.262   0.733
E D  1DVFC                 70    0.401  57.755   0.578   0.003   0.718   0.279
B Y  1DVFC                 71    0.059  12.715  -0.038   0.001   0.900   0.099
B S  1DVFC                 72    0.261  30.648   0.939   0.001   0.900   0.099
B L  1DVFC                 73    0.031   5.713   0.317   0.001   0.959   0.040
E T  1DVFC                 74    0.298  41.374   1.023   0.001   0.959   0.040
B I  1DVFC                 75    0.076  14.079   0.384   0.002   0.816   0.182
E S  1DVFC                 76    0.462  54.135   0.407   0.004   0.514   0.481
E N  1DVFC                 77    0.528  77.328  -0.388   0.005   0.262   0.733
B L  1DVFC                 78    0.138  25.249   0.079   0.004   0.138   0.858
E E  1DVFC                 79    0.453  79.104   0.616   0.018   0.088   0.893
E Q  1DVFC                 80    0.433  77.387  -0.613   0.018   0.088   0.893
E E  1DVFC                 81    0.544  95.002  -0.806   0.053   0.043   0.903
B D  1DVFC                 82    0.241  34.685  -0.775   0.052   0.084   0.864
B I  1DVFC                 83    0.238  44.030   0.066   0.066   0.296   0.638
B A  1DVFC                 84    0.056   6.149  -0.144   0.021   0.756   0.223
B T  1DVFC                 85    0.175  24.259   0.850   0.018   0.846   0.136
B Y  1DVFC                 86    0.041   8.869   1.102   0.018   0.846   0.136
B F  1DVFC                 87    0.060  12.042   1.066   0.021   0.756   0.223
B C  1DVFC                 88    0.020   2.850   1.109   0.021   0.756   0.223
B Q  1DVFC                 89    0.040   7.144  -0.241   0.023   0.655   0.322
B Q  1DVFC                 90    0.110  19.682  -0.096   0.021   0.451   0.528
B G  1DVFC                 91    0.250  19.699  -0.575   0.020   0.205   0.775
E N  1DVFC                 92    0.540  79.056  -1.781   0.018   0.047   0.935
E T  1DVFC                 93    0.430  59.710  -1.995   0.018   0.047   0.935
E L  1DVFC                 94    0.359  65.660  -1.030   0.018   0.088   0.893
E P  1DVFC                 95    0.432  61.230   0.030   0.020   0.205   0.775
B W  1DVFC                 96    0.211  50.745   0.392   0.021   0.451   0.528
B T  1DVFC                 97    0.134  18.558  -1.188   0.023   0.655   0.322
B F  1DVFC                 98    0.118  23.663  -0.814   0.004   0.616   0.381
B G  1DVFC                 99    0.122   9.617  -3.093   0.005   0.262   0.733
E G  1DVFC                100    0.426  33.495  -0.618   0.004   0.085   0.910
E G  1DVFC                101    0.312  24.547  -1.886   0.004   0.197   0.799
B T  1DVFC                102    0.159  21.984  -0.127   0.004   0.420   0.576
E K  1DVFC                103    0.417  85.715   1.593   0.001   0.900   0.099
B L  1DVFC                104    0.112  20.489   0.146   0.001   0.959   0.040
E E  1DVFC                105    0.344  60.184   0.994   0.001   0.900   0.099
B I  1DVFC                106    0.152  28.083  -0.001   0.001   0.900   0.099
E K  1DVFC                107    0.673 138.498   1.479   0.005   0.262   0.733

Explain the output.  Go back.
```

---
